# The protein deficit myth

**DOI:** 10.11606/s1518-8787.2025059006669

**Published:** 2025-06-05

**Authors:** Ricardo Abramovay, Nadine Marques Nunes-Galbes, Fernanda Helena Marrocos-Leite, Eduardo Augusto Fernandes Nilson, Maria Laura da Costa Louzada

**Affiliations:** I Universidade de São Paulo Instituto de Estudos Avançados São Paulo SP Brasil Universidade de São Paulo. Instituto de Estudos Avançados. São Paulo, SP, Brasil; II Universidade de São Paulo Faculdade de Saúde Pública Cátedra Josué de Castro de Sistemas Alimentares Saudáveis e Sustentáveis São Paulo SP Brasil Universidade de São Paulo. Faculdade de Saúde Pública. Cátedra Josué de Castro de Sistemas Alimentares Saudáveis e Sustentáveis. São Paulo, SP, Brasil; III Fundação Oswaldo Cruz Brasília DF Brasil Fundação Oswaldo Cruz. Fiocruz Brasília. Programa de Alimentação, Nutrição e Cultura. Brasília, DF, Brasil; IV Universidade de São Paulo Faculdade de Saúde Pública Departamento de Nutrição São Paulo SP Brasil Universidade de São Paulo. Faculdade de Saúde Pública. Departamento de Nutrição. São Paulo, SP, Brasil

**Keywords:** Protein Requirement, Recommended Dietary Allowances, Formulated Food, Kwashiorkor, Population

## Abstract

Epidemiological data shows that the consumption of animal-based foods in high-income countries is excessive and harmful to health. But the association between poverty and protein deficiency is frequent, both in scientific literature and in the documents of multilateral organizations. There is a conceptual trap in this link, which consists of focusing on one nutrient and not on the whole dietary pattern. In 1974, in a text that has become a classic of nutrition science, Donald McLaren has already highlighted the mistake made by multilateral development organizations in focusing their efforts on protein supply—often in industrialized forms—without considering that, in most cases, once energy needs are met, protein deficiency is unlikely to occur. Data from the 2017–2018 Consumer Expenditure Survey helps to dispel this myth: even among the poorest 20% of the Brazilian population, the proportion of those with insufficient protein intake is tiny.

## INTRODUCTION

An essay published in The Lancet, which marked nutrition science in the second half of the 20th century, turned 50 in 2024 and is still relevant today. The author, Donald McLaren, in an article entitled The Great Protein Fiasco (July 13, 1974)^1^, denounced the formation of a Protein Advisory Group by the United Nations. This group was intended to assist the World Health Organization (WHO) in advising the Food and Agriculture Organization of the United Nations (FAO) and the United Nations Children's Fund (UNICEF) on the safety and suitability of new sources of protein to fill the so-called protein gap, in reference to the supposed deficiency in the intake of this macronutrient. The FAO went so far as to characterize the second half of the 20th century as the "age of protein"^1^.

The topic remains highly relevant in at least two dimensions. The first is the misunderstanding of the very idea of protein deficit, which, according to the view of some of the most important researchers working with United Nations agencies since the 1930s, was the most important feature of child malnutrition in poor countries. McLaren opposes this idea by demonstrating that, apart from exceptional situations in which consumption is reduced to a few foods such as yams or cassava, for example, which have a low protein content, in conditions of energy sufficiency, there is a high probability that there will also be protein sufficiency.

The second dimension of McLaren's critique concerns the construction of the idea that, to fill the supposed protein deficit, the most appropriate thing to do would be to distribute powdered milk (a product of which, not by chance, the United States became surplus from the 1950s onwards) and other industrial formulations rich in protein. Another emblematic case includes the intensification of livestock production by colonial veterinary services in the United Kingdom to increase the consumption of dairy products in populations affected by Kwashiorkor^2^. Such "nutritional concerns" were intrinsically linked to the economic interests of exporters in the Global North.

In his article, McLaren denounces the abandonment of locally available vegetable protein sources in favor of industrially manufactured mixtures made from the abundant North American crops of soy, corn, wheat, and milk. These industrial products were unaffordable for the poorest populations. Some of the products mentioned by McLaren (such as Incaparina or Vitasoy) are still on the market today, without having made any significant contribution to combating hunger in lower-income countries^1^.

The myth of insufficient protein intake (of which the population groups in the worst socio-economic conditions would be the main "victims") remains to this day and has received new support due to the environmental problems linked to the supply of animal foods^3^ as a source of protein. On the one hand, there are many estimates that the demand for meat will increase dramatically with the progressive increase in population and income^4^. On the other hand, meeting this demand comes with huge and growing environmental costs^5^. Against this backdrop, reasoning is gaining momentum that various forms of laboratory-produced proteins^6^ and ultra-processed plant-based products^7^ would be the only way to fill the supposed protein deficit and preserve ecosystem services which, to date, have been sacrificed both by methane emissions from cattle farming and by the socio-environmental consequences of industrial poultry and pig farming, whose gigantic use of antibiotics is at the root of antimicrobial resistance, one of the WHO's most important concerns today^5^.

However, there is a fundamental flaw in this reasoning. If the criteria for this explosive forecast focus on market demand, it is clear that supply will have to increase very significantly. But when it comes to food, it's important to compare the market criteria with the real needs of the human body, which, if considered unlimited, will of course have negative consequences for health^8^. In this sense, what current data shows is that, much more than offering proteins or other nutrients, often through industrial food supplementation or the development of highly publicized "functional" formulas, the greatest challenge of contemporary food is to broaden its diversification, reducing the participation of animal products and ultra-processed foods, and increasing the presence of *fresh* and minimally processed fruits, vegetables and legumes from biodiversity and local cultural, agricultural and culinary traditions^9^.

Adjusting the supply of animal products to the real metabolic and physiological needs of the world's population paves the way for production techniques in which the concentration of animals in the same space is reduced and appropriate management and hygiene practices are expanded^10^, which makes it possible to consistently reduce the use of antibiotics, as is already being done in several European countries^11^. Similarly, the regenerative rearing of cattle on pasture, with moderate intensification techniques and a low degree of confinement, makes it possible to adapt consumption to production models that preserve ecosystem services and animal welfare^12^.

An important question arises here: doesn't this dietary adjustment guideline, which focuses on diversity rather than protein content, underestimate the importance of proteins and, above all, the supposed need to pay special attention to the consumption of proteins by the lower-income population groups, who have the greatest potential for insufficiency?

It's worth considering the myth that accompanies the protein deficit: the myth of the increased need for protein. Based on scientific claims about their various physiological functions^13^, especially and more recently those related to promoting the feeling of satiety and gaining muscle mass^14^, proteins have achieved the status of a "virtuous macronutrient" throughout history, having been highlighted at different times, such as the one we are currently experiencing^15^.

In view of this, over the last 175 years, the recommendations for adequate protein intake for individuals have undergone significant variations, surpassing the mark of 2.0 g/kilogram of weight/day in the second half of the 19th century and stabilizing in the range of 0.6 to 0.8 g/kilogram of weight/day in recent decades^14,15^. Translated into population recommendations, these amounts are equivalent to between 10% and 15% of total daily energy intake^13^.

The myth of the increased need for protein has permeated the popular imagination - including health professionals - to such an extent that high protein intake is now understood and promoted as part of a healthy lifestyle^15,16^. This is reflected in the fact that around 65% of the adult population in the United States takes protein content into account when buying food and drink, as part of a conscious effort to increase their intake of this nutrient, following the logic of ‘the more, the better’^14^.

This perception has been cultivated by multilateral organizations, as discussed by McLaren, and has been conveniently prevalent in the productive sector and reinforced by it, especially among livestock companies and in the plant-based protein food industry. However, the progressive increase in protein intake cannot be understood as risk-free and can have adverse effects on the metabolic functions of the liver, kidneys, pancreas, and even muscle tissue itself^14^. Although there are not many studies on this subject - which, once again, may have more to do with economic interests than health -, a recent study identified an increase in cardiovascular risk associated with protein intake of more than 22% of total daily energy^16^.

To address the question posed above, about a possible underestimation of the importance of protein in the diet, the analysis of the results of the most recent Consumer Expenditure Survey (POF) of the Brazilian population, carried out between 2017 and 2018 and published in 2020, gives a counterintuitive answer, as described below.

### The Case of Brazil

Data from the 2017-2018 POF on food consumption of individuals aged ≥ 10 years, collected through 24-hour food records on two non-consecutive days, were used to estimate food and protein intake in a representative sample of the Brazilian population (n = 46,164)^17^.

Brazil is internationally recognized not only as one of the world's largest producers and exporters of beef, poultry, and pork, but also as one of the main global consumers of these foods. Between 1990 and 2015, Brazil was the country in the world where *per capita* consumption of beef and poultry increased the most^18^.

What is surprising is that, in the distribution of this consumption by income quintiles, the average protein intake exceeds what is necessary to fulfill human metabolic needs in all income quintiles, i.e. beyond the 10 to 15% recommended by the WHO^13^, the contribution of proteins to total energy is around 18%, as shown in Table 1.

**Table 1:** Contribution (%) of energy from protein to the total daily energy consumed by the Brazilian population aged ≥ 10 years by per capita household income quintile. 2017–2018 POF.

Income quintiles	Average	95%CI
1	18.61	18.32–18.90
2	18.22	17.99–18.45
3	18.24	18.02–18.46
4	18.27	17.92–18.61
5	18.37	18.14–18.60

95%CI: 95% confidence interval.

Source: Consumer Expenditure Survey 2017-2018 (n = 46,164).

Considering that the POF data are from 2017-2018, it's likely that the situation will have deteriorated during Jair Bolsonaro's administration (from 2019 to 2022), when the country will once again be on the hunger map. But even so, it is important to note that in all income groups, including the poorest 20% of the Brazilian population (first income quintile), the deficit in protein intake did not reach 3% (Table 2), considering the same WHO recommendations^13^.

**Table 2:** Prevalence of individuals aged ≥ 10 years consuming less than 10% of total daily energy from protein by *per capita* household income quintile.

Income quintiles	Average	95%CI
1	2.60	2.17–3.13
2	3.46	2.75–4.35
3	2.99	2.44–3.66
4	3.19	2.39–4.25
5	2.39	1.90–3.00

95%CI: 95% confidence interval.

Source: Consumer Expenditure Survey 2017–2018 (n = 46,164).

This reflects a high consumption of all types of meat and other protein-rich foods (for example, legumes - including different varieties of beans) in all socio-economic strata (Table 3). On the other hand, the disparity in access to other healthy and diversified foods can be seen, for example, in the average consumption of fruit and vegetables across the income quintiles, from 3.4% of total calorie intake in the lowest income quintile to 6.4% in the highest income quintile (Table 3).

**Table 3:** Average consumption (in g/day) of food groups by *per capita* household income quintiles (Brazilian population aged ≥ 10 years). 2017–2018 POF.

Income quintiles		Average	95%CI
Beef			
	1	55.42	51.71–59.13
	2	56.63	53.55–59.70
	3	61.46	58.44–64.49
	4	65.45	62.56–68.34
	5	63.83	60.57–67.09
Poultry			
	1	52.41	49.32–55.50
	2	57.60	54.35–60.84
	3	52.42	49.07–55.76
	4	50.89	47.14–54.64
	5	46.27	43.37–49.17
Pork			
	1	15.08	12.67–17.49
	2	16.87	14.51–19.23
	3	17.02	14.79–19.24
	4	17.74	15.48–20.01
	5	13.59	11.76–15.42
Beans			
	1	7.73	7.42–8.04
	2	7.35	7.08–7.62
	3	6.65	6.38–6.92
	4	5.77	5.53–6.01
	5	4.16	3.94–4.37
Fruits, vegetables, and greens			
	1	3.39	3.24–3.54
	2	4.14	3.95–4.32
	3	4.66	4.46–4.86
	4	5.31	5.10–5.51
	5	6.43	6.17–6.70

95%CI: 95% confidence interval.

Source: Consumer Expenditure Survey 2017–2018 (n = 46,164).

These findings point to the need for the focus that has historically been so much on protein consumption – reflecting both concern about a supposedly insufficient intake and the promotion of an increasing intake – to shift to what is truly lacking in Brazilians’ diets: fruit, vegetables, and greens. According to the Telephone-based Surveillance of Risk and Protective Factors for Chronic Diseases (Vigitel)^19^, which is also nationally representative, no less than 78.6% of adults living in Brazilian state capitals did not meet the WHO recommendation for fruit, vegetable and greens consumption (400 g/day/person) in 2023^20^.

## CONCLUSION

The findings from the analysis of the Brazilian population's consumption data are fundamental for rethinking the relationship between food systems, climate, and biodiversity. If the human metabolic needs met by foods that are currently vectors of socio-environmental destruction are lower than is usually estimated, this increases the possibility of protecting and promoting biodiversity^5^, for example by improving and expanding regenerative production practices, while benefiting human health.
