# Erratum

**DOI:** 10.11606/s1518-8787.2025059006669err

**Published:** 2025-09-08

In **The protein deficit myth**, with DOI number https://doi.org/10.11606/s1518-8787.2025059006669, published in the journal Revista de Saúde Pública, v. 59, e21 on page 5,

**Where it reads:**

**It should read:**

**Table 3:** Average consumption (in g/day) of food groups by *per capita* household income quintiles (Brazilian population aged ≥ 10 years). 2017–2018 POF.

Income quintiles	Average	95%CI
Beef		
1	55.42	51.71–59.13
2	56.63	53.55–59.70
3	61.46	58.44–64.49
4	65.45	62.56–68.34
5	63.83	60.57–67.09
Poultry		
1	52.41	49.32–55.50
2	57.60	54.35–60.84
3	52.42	49.07–55.76
4	50.89	47.14–54.64
5	46.27	43.37–49.17
Pork		
1	15.08	12.67–17.49
2	16.87	14.51–19.23
3	17.02	14.79–19.24
4	17.74	15.48–20.01
5	13.59	11.76–15.42
Beans		
1	7.73	7.42–8.04
2	7.35	7.08–7.62
3	6.65	6.38–6.92
4	5.77	5.53–6.01
5	4.16	3.94–4.37
Fruits, vegetables, and greens		
1	3.39	3.24–3.54
2	4.14	3.95–4.32
3	4.66	4.46–4.86
4	5.31	5.10–5.51
5	6.43	6.17–6.70

95%CI: 95% confidence interval.

Source: Consumer Expenditure Survey 2017–2018 (n = 46,164).

**Table 3:** Average consumption (in g/daya or % of daily energyb) of food groups by *per capita* household income quintiles (Brazilian population aged ≥ 10 years). 2017–2018 POF.

Income quintiles	Average	95%CI	
Beef^a^			
1	55.42	51.71	59.13
2	56.63	53.55	59.70
3	61.46	58.44	64.49
4	65.45	62.56	68.34
5	63.83	60.57	67.09
Poultry^a^			
1	52.41	49.32	55.50
2	57.60	54.35	60.84
3	52.42	49.07	55.76
4	50.89	47.14	54.64
5	46.27	43.37	49.17
Pork^a^			
1	15.08	12.67	17.49
2	16.87	14.51	19.23
3	17.02	14.79	19.24
4	17.74	15.48	20.01
5	13.59	11.76	15.42
Beans^b^			
1	7.73	7.42	8.04
2	7.35	7.08	7.62
3	6.65	6.38	6.92
4	5.77	5.53	6.01
5	4.16	3.94	4.37
Fruits, vegetables, and greens^b^			
1	3.39	3.24	3.54
2	4.14	3.95	4.32
3	4.66	4.46	4.86
4	5.31	5.10	5.51
5	6.43	6.17	6.70

95%CI: 95% confidence interval.

Source: Consumer Expenditure Survey 2017–2018 (n = 46.164).

^a^ Food groups for which average consumption is presented in g/day.

^b^ Food groups for which average consumption is presented in % of daily energy.

