# Supplementary material for: The protein deficit myth
Source: Rev Saude Publica. 2025 Jun 5;59:e21. doi: 10.11606/s1518-8787.2025059006669 (PMC12204648; doi:10.11606/s1518-8787.2025059006669)
Supplement: Translation

# Translation: O mito do déficit proteico

**Authors:** Ricardo Abramovay, Nadine Marques Nunes-Galbes, Fernanda Helena Marrocos-Leite, Eduardo Augusto Fernandes Nilson, Maria Laura da Costa Louzada

**Affiliations:** I São Paulo SP Brasil Universidade de São Paulo. Instituto de Estudos Avançados. São Paulo, SP, Brasil; II São Paulo SP Brasil Universidade de São Paulo. Faculdade de Saúde Pública. Cátedra Josué de Castro de Sistemas Alimentares Saudáveis e Sustentáveis. São Paulo, SP, Brasil; III Brasília DF Brasil Fundação Oswaldo Cruz. Fiocruz Brasília. Programa de Alimentação, Nutrição e Cultura. Brasília, DF, Brasil; IV São Paulo SP Brasil Universidade de São Paulo. Faculdade de Saúde Pública. Departamento de Nutrição. São Paulo, SP, Brasil

**Keywords:** Necessidade Proteica, Recomendações Nutricionais, Alimentos Formulados, Kwashiorkor, População

## Abstract

Dados epidemiológicos apontam que o consumo de alimentos de origem animal nos países de alta renda é excessivo e prejudicial à saúde. Mas é frequente, tanto na literatura científica como nos documentos das organizações multilaterais, a associação entre pobreza e carência de proteínas. Há uma armadilha conceitual neste vínculo, que consiste em concentrar a atenção em um nutriente e não no conjunto do padrão alimentar. Em 1974, num texto que se tornou um clássico da ciência da nutrição, Donald McLaren já mostrava o erro das organizações multilaterais de desenvolvimento em focar seus esforços na oferta de proteínas (inclusive sob formas industrializadas) sem levar em conta que, com raras exceções, quando se alcança suficiência energética, dificilmente haverá déficit proteico. Dados da Pesquisa de Orçamentos Familiares de 2017 a 2018 ajudam a desfazer esse mito: mesmo entre os 20% mais pobres da população brasileira, é ínfima a proporção dos que apresentam ingestão proteica insuficiente.

## INTRODUÇÃO

Um ensaio publicado pelo *The Lancet*, que marcou a ciência da nutrição na segunda metade do século XX, completou 50 anos em 2024 e se mantém atual até os dias de hoje. O autor, Donald McLaren, no artigo intitulado *The Great Protein Fiasco* (13/07/1974)^1^, denunciava a formação, pelas Nações Unidas, de um Grupo Consultivo sobre Proteínas (ou *Protein Advisory Group,* em inglês). Tal grupo era voltado a auxiliar a Organização Mundial da Saúde (OMS) no aconselhamento à Organização das Nações Unidas para Alimentação e Agricultura (FAO) e à Fundo das Nações Unidas para a Infância (Unicef) sobre a segurança e adequação de novas fontes de proteínas para preencher o chamado *protein gap*, que podemos compreender como lacuna proteica, em referência à suposta deficiência de ingestão deste macronutriente. A FAO chegava ao ponto de caracterizar a segunda metade do século XX como a "era da proteína"^1^.

O tema mantém toda sua atualidade sob ao menos duas dimensões. A primeira está no equívoco da própria ideia de déficit proteico, que, segundo a visão de alguns dos mais importantes pesquisadores que atuavam junto às agências das Nações Unidas, desde os anos 1930, era o traço mais importante da desnutrição infantil nos países pobres. McLaren se contrapõe a esta ideia, ao demonstrar que, salvo em situações excepcionais em que o consumo se reduz a alguns poucos alimentos como inhame ou mandioca, por exemplo, que apresentam baixo teor de proteínas, em condições de suficiência energética, é alta a probabilidade de que haja igualmente suficiência proteica.

A segunda dimensão da denúncia de McLaren diz respeito à construção da ideia de que, para preencher o suposto déficit proteico, o mais adequado seria a farta distribuição de leite em pó (produto do qual, não por acaso, os Estados Unidos tornavam-se excedentários, a partir dos anos 1950) e de outras formulações industriais ricas em proteínas. Outro caso emblemático inclui a intensificação da produção pecuária por serviços veterinários coloniais no Reino Unido a fim de aumentar o consumo de produtos lácteos em populações acometidas por Kwashiorkor^2^. Tais "preocupações nutricionais" estavam intrinsicamente ligadas aos interesses econômicos dos exportadores do Norte Global.

McLaren, em seu artigo, denuncia o abandono de fontes vegetais proteicas localmente disponíveis em benefício de misturas fabricadas industrialmente a partir de produtos das abundantes safras norte-americanas de soja, milho, trigo e também de leite. Estes produtos industriais tinham preços inacessíveis às populações mais pobres. Alguns dos produtos citados por McLaren (como Incaparina ou Vitasoy) estão no mercado até hoje, sem que tenham oferecido qualquer contribuição expressiva no combate à fome nos países pobres^1^.

O mito da ingestão proteica insuficiente (da qual os grupos populacionais em piores condições socioeconômicas seriam as principais "vítimas") permanece até hoje e recebeu novas frentes de apoio em função dos problemas ambientais ligados à oferta de alimentos de origem animal^3^ fonte de proteínas. Por um lado, não são poucas as estimativas de que a demanda por carnes vai aumentar de forma estrondosa com o aumento progressivo da população e da renda^4^. Por outro, o atendimento a esta demanda traz custos ambientais gigantescos e crescentes^5^. A partir deste cenário, vem ganhando força o raciocínio de que formas variadas de proteínas produzidas em laboratório^6^ e produtos ultraprocessados *plant-based*^7^ seriam a única chance para se preencher o suposto déficit proteico e preservar serviços ecossistêmicos que, até o presente momento, vêm sendo sacrificados tanto pelas emissões de metano da criação de gado bovino, como pelas consequências socioambientais das criações industriais de aves e suínos, cujo gigantesco uso de antibióticos está na origem da resistência antimicrobiana, uma das mais importantes preocupações da OMS na atualidade^5^.

Contudo, existe um vício fundamental neste raciocínio. Se os critérios para esta previsão explosiva se concentrarem nas demandas de mercado, é claro que a oferta terá que aumentar de forma muito expressiva. Mas, tratando-se de alimentos, é importante cotejar o critério de mercado com as reais necessidades do organismo humano, que, se consideradas como ilimitadas, trazem, é claro, consequências negativas para a saúde^8^. Neste sentido, o que os dados atuais mostram é que, muito mais do que oferecer proteínas ou outros nutrientes, muitas vezes a partir da suplementação industrial de alimentos ou pela elaboração de fórmulas "funcionais" altamente publicizadas, o maior desafio da alimentação contemporânea está em ampliar sua diversificação, reduzindo a participação de produtos de origem animal e de ultraprocessados, e ampliando a presença de frutas, verduras e legumes *in natura* e minimamente processados provenientes da biodiversidade e de tradições culturais, agropecuárias e culinárias locais^9^.

A adequação da oferta de produtos de origem animal às reais necessidades metabólicas e fisiológicas da população mundial abre caminho para técnicas produtivas em que se reduz a concentração de animais num mesmo espaço e se amplia práticas de manejo e higiene adequados^10^, o que permite diminuir de maneira consistente, como já se faz em vários países europeus, o uso de antibióticos^11^. Da mesma forma, a criação regenerativa de gado bovino a pasto, com técnicas de intensificação moderada e baixo grau de confinamento, permite adequar o consumo a modelos produtivos que preservam serviços ecossistêmicos e bem-estar animal^12^.

Aqui emerge uma importante questão: será que esta orientação de ajuste dietético com enfoque na diversidade, e não no teor de proteínas, não subestima a importância das proteínas e sobretudo da suposta necessidade de especial atenção ao consumo de proteínas pelas camadas populacionais de menor renda, com maior potencial de insuficiência?

Cabe situar tal questão a partir da construção do mito que acompanha o do déficit proteico: o mito da necessidade aumentada de proteínas. Com base nas alegações científicas a respeito de suas variadas funções fisiológicas^13^, em especial e mais recentemente aquelas relacionadas à promoção da sensação de saciedade e ao ganho de massa muscular^14^, as proteínas alcançaram, ao longo da história, um status de "macronutriente virtuoso", tendo sido colocadas em evidência em diferentes momentos, como o que vivemos atualmente^15^.

Em vista disso, ao longo dos últimos 175 anos, as recomendações de ingestão adequada de proteínas para indivíduos sofreram importantes variações, tendo superado a marca de 2,0 g/quilo de peso/dia na segunda metade do século XIX, e se estabilizando na faixa dos 0,6 a 0,8 g/quilo de peso/dia nas últimas décadas^14,15^. Traduzidas para recomendações populacionais, essas quantidades equivalem a uma faixa de 10% a 15% do total de energia ingerida diariamente^13^.

O mito da necessidade aumentada de proteínas passou a permear o imaginário popular – incluindo aí os profissionais de saúde – de tal modo que a elevada ingestão de proteínas é, atualmente, compreendida e promovida como parte de um estilo de vida saudável^15,16^. Isso se reflete no dado de que cerca de 65% da população adulta dos Estados Unidos leva em conta o teor de proteínas ao comprar alimentos e bebidas, como parte de um esforço consciente de aumento da ingestão deste nutriente, seguindo a lógica de ‘quanto mais, melhor’^14^.

Tal percepção foi cultivada pelas organizações multilaterais, como discutido por McLaren, e vem sendo convenientemente prevalente no setor produtivo e reforçada por ele, em especial entre pecuaristas, frigoríficos e na indústria de alimentos *plant-based* fonte de proteínas. Entretanto, o aumento progressivo da ingestão de proteínas não pode ser compreendido como isento de risco, podendo apresentar efeitos adversos sobre funções metabólicas do fígado, rins, pâncreas e até mesmo do próprio tecido muscular^14^. Embora os estudos a esse respeito não sejam abundantes – o que, mais uma vez, pode ter mais relação com interesses econômicos do que de saúde, propriamente – um estudo recente identificou aumento do risco cardiovascular associado à ingestão proteica superior a 22% do total de energia diário^16^.

No intuito de endereçar a questão colocada anteriormente, sobre uma possível subestimação da importância das proteínas na alimentação, a análise dos resultados da mais recente Pesquisa de Orçamentos Familiares (POF) da população brasileira, realizada entre 2017 e 2018 e publicada em 2020, responde de maneira contraintuitiva, conforme descrito abaixo.

### O Caso do Brasil

Dados da POF 2017–2018 de consumo alimentar de indivíduos com idade ≥ 10 anos, coletados por meio de registros alimentares de 24 h em dois dias não consecutivos, foram utilizados para estimar a ingestão de alimentos e de proteína em amostra representativa da população brasileira (n = 46.164)^17^.

O Brasil é reconhecido internacionalmente não apenas como um dos maiores produtores e exportadores mundiais de carne bovina, de aves e de suínos, mas também como um dos principais consumidores globais destes alimentos. Entre 1990 e 2015, o Brasil foi o país do mundo em que mais aumentou o consumo *per capita* de carne bovina e de aves^18^.

O que surpreende é que, na distribuição deste consumo por faixa de renda, a ingestão proteica média supera o necessário ao preenchimento das necessidades metabólicas humanas em todas as faixas de renda, ou seja, para além dos 10 a 15% recomendados pela OMS^13^, a contribuição das proteínas para o total de energia gira em torno de 18%, como mostra a Tabela 1.

**Tabela 1:** Contribuição (%) da energia advinda de proteínas para o total diário de energia consumida pela população brasileira de ≥ 10 anos por quintos de renda familiar *per capita*. POF 2017–2018.

Quintos de renda	Média	IC95%
1	18,61	18,32–18,90
2	18,22	17,99–18,45
3	18,24	18,02–18,46
4	18,27	17,92–18,61
5	18,37	18,14–18,60

IC95%: intervalo de confiança de 95%.

Fonte: Pesquisa de Orçamentos Familiares 2017–2018 (n = 46.164).

Considerando que os dados da POF são de 2017–2018, é provável que a situação tenha se deteriorado durante o governo de Jair Bolsonaro (de 2019 a 2022), quando o país voltou a figurar no mapa da fome. Mas, ainda assim, é importante constatar que, em todas as faixas de renda, incluindo os 20% mais pobres da população brasileira (primeiro quinto de renda), o déficit na ingestão de proteínas não chegava a 3% (Tabela 2), considerando as mesmas recomendações da OMS^13^.

**Tabela 2:** Prevalência de pessoas de ≥ 10 anos com consumo inferior a 10% do total diário de energia advindo de proteínas por quintos de renda familiar *per capita*.

Quintos de renda	Média	IC95%
1	2,60	2,17–3,13
2	3,46	2,75–4,35
3	2,99	2,44–3,66
4	3,19	2,39–4,25
5	2,39	1,90–3,00

IC95%: intervalo de confiança de 95%.

Fonte: Pesquisa de Orçamentos Familiares 2017–2018 (n = 46.164).

Este dado reflete um alto consumo de todos os tipos de carne e outros alimentos ricos em proteínas (por exemplo, as leguminosas – incluindo as diferentes variedades de feijões) em todos os estratos socioeconômicos (Tabela 3). Em contrapartida, a disparidade no acesso a outros alimentos saudáveis e diversificados se revela, por exemplo, a partir da média do consumo de frutas, legumes e verduras ao longo dos quintos de renda, passando de 3,4% do total de calorias ingeridas no quinto de menor renda para 6,4% no quinto de maior renda (Tabela 3).

**Tabela 3:** Consumo médio (em g/dia) de grupos alimentares por quintos de renda familiar *per capita* (população brasileira de ≥ 10 anos). POF 2017–2018.

Quintos de renda		Média	IC95%
Carne bovina			
	1	55,42	51,71–59,13
	2	56,63	53,55–59,70
	3	61,46	58,44–64,49
	4	65,45	62,56–68,34
	5	63,83	60,57–67,09
Aves			
	1	52,41	49,32–55,50
	2	57,60	54,35–60,84
	3	52,42	49,07–55,76
	4	50,89	47,14–54,64
	5	46,27	43,37–49,17
Carne suína			
	1	15,08	12,67–17,49
	2	16,87	14,51–19,23
	3	17,02	14,79–19,24
	4	17,74	15,48–20,01
	5	13,59	11,76–15,42
Feijão			
	1	7,73	7,42–8,04
	2	7,35	7,08–7,62
	3	6,65	6,38–6,92
	4	5,77	5,53–6,01
	5	4,16	3,94–4,37
Frutas, legumes e verduras			
	1	3,39	3,24–3,54
	2	4,14	3,95–4,32
	3	4,66	4,46–4,86
	4	5,31	5,10–5,51
	5	6,43	6,17–6,70

IC95%: intervalo de confiança de 95%.

Fonte: Pesquisa de Orçamentos Familiares 2017–2018 (n = 46.164).

Tais achados apontam para a necessidade de que os olhares, historicamente tão voltados ao consumo de proteínas – refletindo tanto a preocupação com uma suposta ingestão insuficiente, quanto a promoção de uma ingestão crescente - se desloquem para o que se encontra verdadeiramente em falta na alimentação dos brasileiros: as frutas, legumes e verduras. De acordo com a pesquisa Vigilância de Fatores de Risco e Proteção para Doenças Crônicas por Inquérito Telefônico (Vigitel)^19^, que também apresenta representatividade nacional, nada menos que 78,6% dos adultos residentes nas capitais de estados brasileiros não alcançavam a recomendação da OMS para consumo de frutas, legumes e verduras (400 g/dia/pessoa) em 2023^20^.

## CONCLUSÃO

Os achados evidenciados a partir da análise de dados de consumo da população brasileira são fundamentais para repensar a relação entre sistemas alimentares, clima e biodiversidade. Se as necessidades metabólicas humanas supridas por alimentos que hoje são vetores de destruição socioambiental são menores do que habitualmente se estima, isso amplia a possibilidade de proteção e promoção da biodiversidade^5^, a partir, por exemplo, do aprimoramento e da expansão de práticas produtivas regenerativas, beneficiando, ao mesmo tempo, a saúde humana.
