# Supplementary material for: Erratum
Source: Rev Saude Publica. 2025 Sep 8;59:e21err. doi: 10.11606/s1518-8787.2025059006669err (PMC12537014; doi:10.11606/s1518-8787.2025059006669err)
Supplement: Translation

# Translation: Errata

No artigo **O mito do déficit proteico**, com número de DOI https://doi.org/10.11606/s1518-8787.2025059006669, publicado na Revista de Saúde Pública, v. 59, e21 na página 5.

**Onde se lê:**

**Leia-se:**

**Tabela 3:** Consumo médio (em g/dia) de grupos alimentares por quintos de renda familiar *per capita* (população brasileira de ≥ 10 anos). POF 2017–2018.

Quintos de renda	Média	IC95%
Carne bovina		
1	55,42	51,71–59,13
2	56,63	53,55–59,70
3	61,46	58,44–64,49
4	65,45	62,56–68,34
5	63,83	60,57–67,09
Aves		
1	52,41	49,32–55,50
2	57,60	54,35–60,84
3	52,42	49,07–55,76
4	50,89	47,14–54,64
5	46,27	43,37–49,17
Carne suína		
1	15,08	12,67–17,49
2	16,87	14,51–19,23
3	17,02	14,79–19,24
4	17,74	15,48–20,01
5	13,59	11,76–15,42
Feijão		
1	7,73	7,42–8,04
2	7,35	7,08–7,62
3	6,65	6,38–6,92
4	5,77	5,53–6,01
5	4,16	3,94–4,37
Frutas, legumes e verduras		
1	3,39	3,24–3,54
2	4,14	3,95–4,32
3	4,66	4,46–4,86
4	5,31	5,10–5,51
5	6,43	6,17–6,70

IC95%: intervalo de confiança de 95%.

Fonte: Pesquisa de Orçamentos Familiares 2017–2018 (n = 46.164).

**Tabela 3:** Consumo médio (em g/diaa ou % calorias/diab) de grupos alimentares por quintos de renda familiar *per capita* (população brasileira de ≥ 10 anos). POF 2017–2018.

Quintos de renda	Média	IC95%	
Carne bovina^a^			
1	55,42	51,71	59,13
2	56,63	53,55	59,70
3	61,46	58,44	64,49
4	65,45	62,56	68,34
5	63,83	60,57	67,09
Aves^a^			
1	52,41	49,32	55,50
2	57,60	54,35	60,84
3	52,42	49,07	55,76
4	50,89	47,14	54,64
5	46,27	43,37	49,17
Carne suína^a^			
1	15,08	12,67	17,49
2	16,87	14,51	19,23
3	17,02	14,79	19,24
4	17,74	15,48	20,01
5	13,59	11,76	15,42
Feijão^b^			
1	7,73	7,42	8,04
2	7,35	7,08	7,62
3	6,65	6,38	6,92
4	5,77	5,53	6,01
5	4,16	3,94	4,37
Frutas, legumes e verduras^b^			
1	3,39	3,24	3,54
2	4,14	3,95	4,32
3	4,66	4,46	4,86
4	5,31	5,10	5,51
5	6,43	6,17	6,70

IC95%: intervalo de confiança de 95%.

Fonte: Pesquisa de Orçamentos Familiares 2017–2018 (n = 46.164).

^a^ Grupos de alimentos cujo consumo médio é apresentado em g/dia.

^b^ Grupos de alimentos cujo consumo médio é apresentado em % de energia diária.
